# A Case of Anterior Vitreous Displacement of the Intravitreal Dexamethasone Implant (Ozurdex®) in a Pseudophakic Eye

**DOI:** 10.7759/cureus.94264

**Published:** 2025-10-10

**Authors:** Amine Mahdar, Pauline Dubois

**Affiliations:** 1 Ophthalmology Department, Saint Pierre University Hospital, Université Libre de Bruxelles, Brussels, BEL

**Keywords:** anterior migration of ozurdex, anterior vitreous displacement, branch retinal vein occlusion, implant migration, intravitreal dexamethasone implant, macular edema, ocular complications, ozurdex

## Abstract

Macular edema following branch retinal vein occlusion (BRVO) is a common cause of visual impairment. Treatment options include intravitreal anti-vascular endothelial growth factor injections as first-line therapy, followed by a dexamethasone implant (Ozurdex^®^), preservative-free triamcinolone, and macular laser photocoagulation. Here, we present a case of anterior vitreous displacement of the Ozurdex^®^ implant, a rare non-pharmacological complication. An 88-year-old pseudophakic woman with BRVO and macular edema received an intravitreal dexamethasone implant (Ozurdex^®^). After the injection, she reported a rod-like floater. Examination revealed no visual loss or raised intraocular pressure. The implant was located behind the intraocular lens in the anterior vitreous, with stable macular optical coherence tomography and fundus findings. Conservative follow-up was chosen, and the implant dissolved over four months without complications. This rare displacement contrasts with anterior chamber migration, which can be vision-threatening. Anterior vitreous displacement of an Ozurdex^®^ implant in a pseudophakic eye is benign and does not require surgical intervention. A conservative “watch-and-wait” approach with close monitoring is sufficient. Our case emphasizes preventive strategies, including identifying at-risk patients, using a careful injection technique, and performing meticulous slit-lamp evaluation in cases of migration.

## Introduction

The dexamethasone intravitreal implant 700 µg (Ozurdex™, Allergan^®^) is a biodegradable corticosteroid intravitreal implant approved by the Food and Drug Administration for the treatment of diabetic macular edema, macular edema following a branch retinal vein occlusion (BRVO) or central retinal vein occlusion, and posterior segment non-infectious uveitis [1]. It measures 6.0 mm in length and 0.46 mm in diameter and is injected through the pars plana into the vitreous, gradually releasing corticosteroids [2].

Adverse effects can be divided into two categories, namely, pharmacological (glaucoma and steroid-induced cataracts) and non-pharmacological (subconjunctival hemorrhage, anterior chamber migration, vitreous hemorrhage, endophthalmitis, hypotony, rhegmatogenous retinal detachment, and inadvertent implant misplacement - intralenticular, suprachoroidal, or double injection) [3].

Here, we report a case of a non-pharmacological side effect: anterior vitreous displacement of the intravitreal dexamethasone implant (Ozurdex^®^), which has only been described twice in the literature to date: in 2013 by Wai et al. in a phakic patient (without penetration of the posterior lens) who received the implant for BRVO with cystoid macular edema [4], and in 2024 by Banerjee et al. in a pseudophakic patient who received the implant in the context of vitrectomy with silicone oil tamponade for detachment with established proliferative vitreoretinopathy [5].

## Case presentation

**Figure 1:**
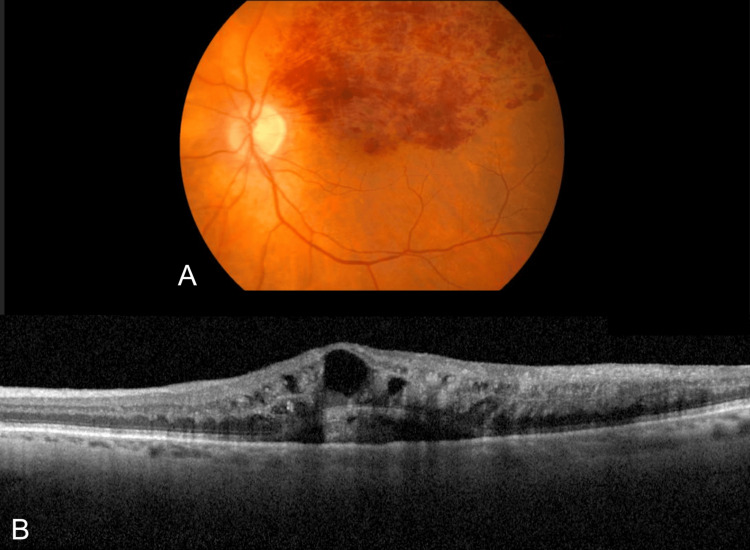
Fundus photograph (A) and macular optical coherence tomography (B) of an 88-year-old patient with a superior branch retinal vein occlusion, hemorrhagic and edematous.

An 88-year-old woman was under follow-up in our department for a superior BRVO, complicated by macular edema (Figure 1). She had a medical history of dyslipidemia. She had no significant ophthalmic history except for uneventful phacoemulsification with intraocular lens implantation in the capsular bag, followed by Nd:YAG laser capsulotomy for posterior capsular opacification in both eyes. She had no history of vitrectomy. The patient had previously undergone eight intravitreal injections of ranibizumab at four-week intervals, resulting in an improvement in best-corrected visual acuity from 20/50 to 20/22. Treatment with a dexamethasone intravitreal implant (Ozurdex^®^) was initiated to target the inflammatory component of the edema and resolve it.

A trained ophthalmologist performed the intravitreal injections at an early stage of surgical experience under controlled aseptic conditions and topical anesthesia. The injection was performed in accordance with the manufacturer’s recommended administration protocol [1]. The long axis of the applicator was maintained parallel to the limbus, and the sclera was engaged at an oblique angle with the needle bevel oriented upward. The needle tip was advanced within the sclera for approximately 1 mm (parallel to the limbus), then redirected toward the center of the globe and advanced until full scleral penetration was achieved and the vitreous cavity was entered.

**Figure 2:**
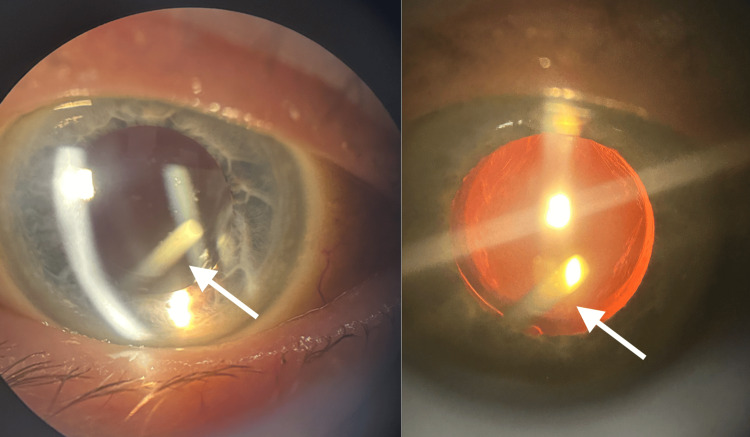
Slit-lamp examination of the patient with narrow beam illumination (A) and retroillumination (B) showing the presence of an Ozurdex® implant (white arrow) located behind the intraocular lens in the anterior vitreous.

The patient presented to the ophthalmic emergency department a few days after her second intravitreal injection of Ozurdex^®^, reporting a rod-like floater in her visual field. This symptom appeared immediately after the injection and was not associated with any decrease in visual acuity. Slit-lamp examination revealed a quiet anterior chamber, a clear cornea, and the presence of an Ozurdex^®^ implant located behind the intraocular lens in the anterior vitreous (Figure 2).

**Figure 3:**
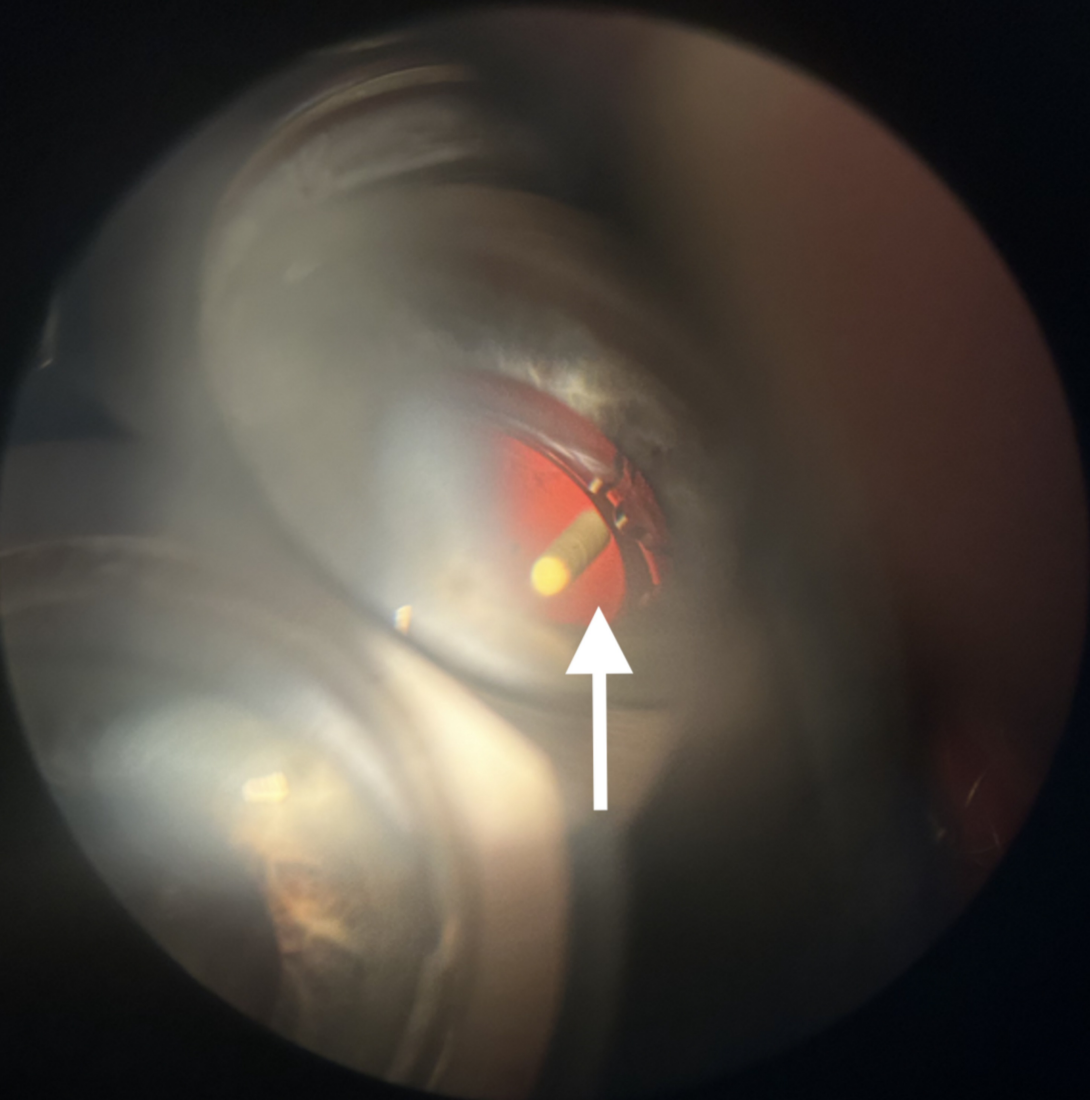
Three-mirror lens examination showing no adhesion between the posterior capsule and the Ozurdex® implant (white arrow).

The anterior capsulorhexis was circular and without notches, and the intraocular lens was well-centered in the capsular bag without tilt. The intraocular pressure was measured at 14 mmHg. The three-mirror lens examination showed no adhesion between the Ozurdex^®^ implant and the posterior capsule that had previously undergone Nd:YAG laser capsulotomy (Figure 3).

Macular optical coherence tomography was stable, as the patient presented only a few days after the Ozurdex^®^ injection, which was too early to observe its therapeutic effect, namely, the resorption of macular edema. Fundus examination showed a clear vitreous with a flat retina. Intraocular pressure remained within normal limits throughout follow-up. A conservative approach with close monitoring was preferred over Nd:YAG vitreolysis or any surgical intervention. The implant remained in the anterior vitreous and gradually dissolved over a period of four months, without causing any anterior or posterior segment complications. The treatment was switched to ranibizumab, and the macular edema resolved after three injections.

## Discussion

In our case, the patient was pseudophakic. She received Ozurdex^®^ for BRVO with cystoid macular edema. She presented to the ophthalmic emergency department a few days after the injection of Ozurdex^®^, reporting a rod-like floater in her visual field, which had appeared immediately after the injection. Specific advice from a vitreoretinal surgeon was sought to determine the necessity of surgery or Nd:YAG vitreolysis. This procedure would have consisted of lysing the vitreous located inferior to the Ozurdex^®^ implant to create a descending tunnel within the vitreous cavity, allowing the implant to settle into the inferior vitreous by gravity. However, this approach is not without risk [6]. First, due to the friable nature of the implant, it could potentially fragment into multiple pieces [7] and fail to alleviate the patient’s visual discomfort. Second, Nd:YAG vitreolysis at energies of 3 to 5 mJ alters the vitreoretinal traction forces, thereby increasing the risk of retinal detachment [6]. As suggested in the cases reported by Wai et al. and Banerjee et al., a conservative approach was preferred, without the need for surgical intervention or Nd:YAG vitreolysis.

To reduce the risk of Ozurdex^®^ migration, it is essential to identify the confirmed risk factors [8]. In our case, the patient was pseudophakic with capsular defects (posterior capsule opacification managed with capsulotomy), a known risk factor for anterior chamber migration of the Ozurdex^®^ implant [8], which can expose the eye to serious complications such as corneal edema, corneal decompensation, and intraocular hypertension [9], and may require prompt removal (using intraocular forceps, viscoelastic expression, or a 20-gauge cannula) [10]. Other risk factors for anterior chamber migration of the Ozurdex^®^ implant include aphakia, previous vitrectomy, iris-claw lenses, iris-sutured intraocular lenses, zonular defects, and various complications associated with prior ocular procedures [9,10].

The anterior vitreous displacement of the Ozurdex^®^ implant should be clearly distinguished from anterior migration into the anterior chamber, which can result in severe corneal damage and may necessitate corneal transplantation [11,12]. Furthermore, the anterior vitreous displacement of the Ozurdex^®^ implant in a pseudophakic eye should also be distinguished from anterior vitreous displacement with penetration of the posterior lens in a phakic eye, a condition that often requires phacoemulsification with implant removal, followed by vitrectomy and the insertion of a sulcus lens [13-15].

Regarding the pathophysiological mechanism of the displacement, Wai et al. suggested retention within the anterior hyaloid fossa (in a phakic eye) [4], while Banerjee et al. proposed that a combination of oil buoyancy force and a possible weak adhesion between the implant surface and the posterior lens capsule resulted in its transient anomalous position (in a pseudophakic silicone oil-filled eye) [5]. In our case, the patient did not receive silicone oil, and a three-mirror lens examination did not reveal any adhesion between the implant and the posterior capsule that had previously undergone Nd:YAG laser capsulotomy. Therefore, Wai et al.’s hypothesis [4] of retention within the anterior hyaloid fossa may plausibly explain the mechanism observed in our case.

In our case, the procedure leading to the displacement of the Ozurdex^®^ implant was performed by a trained ophthalmologist at an early stage of surgical experience, following the standard technique recommended by the manufacturer [1]. Several authors have suggested modifying the injection technique by adopting the “bevel-down and toward-the-wall” approach to direct the implant toward the peripheral retina and the posterior segment of the globe [16], while others have proposed injecting a viscoelastic substance into the Ozurdex^®^ implant needle to reduce the ejection velocity by nearly 87.87% in vitrectomized eyes [17]. Furthermore, it has been demonstrated that administering the Ozurdex^®^ implant over a three-second period significantly reduces its velocity and insertion force, thereby lowering the risk of retinal tears and vitreous hemorrhage [18].

## Conclusions

This article highlights key clinical implications. To minimize the risk of Ozurdex^®^ displacement or migration, at-risk patients should be carefully identified, and the implant should be delivered over a three-second period using the “bevel-down and toward-the-wall” technique. In addition, management depends entirely on the precise localization of the implant, which must be assessed through a meticulous slit-lamp examination. Unlike anterior chamber migration, an anterior vitreous displacement in a pseudophakic eye, as illustrated in our case, is generally benign and does not lead to complications. In such circumstances, no surgical intervention or repositioning maneuver is required. A conservative “watch-and-wait” approach with close monitoring is sufficient, allowing the implant to dissolve spontaneously over time.
